# Breast Hemangioma Diagnosed in a Male Patient: A Case Report

**DOI:** 10.7759/cureus.78390

**Published:** 2025-02-02

**Authors:** Tsion Assaye, Luma Abunimer, Nabil Calisi, Di Ai

**Affiliations:** 1 Transitional Year Residency Program, Wellstar Kennestone Regional Medical Center, Marietta, USA; 2 Department of Radiology and Imaging Science, Emory University School of Medicine, Atlanta, USA; 3 Department of Pathology and Laboratory Medicine, University of Texas Health Science Center at Houston, Houston, USA

**Keywords:** angiosarcoma, breast hemangioma, breast sonography, breast ultrasound, core needle biopsy, mammography

## Abstract

Vascular tumors rarely occur in the breast, with hemangiomas being the most common benign form. The vast majority of these lesions occur in women and are clinically asymptomatic and non-palpable on exam, often detected as incidental findings during routine mammography. This report describes and discusses the case of a symptomatic breast hemangioma diagnosed in a 69-year-old male patient with no pertinent medical history, who presented to his primary care provider with complaints of vague left-sided chest wall pain to palpation. Mammography revealed an isodense and circumscribed oval-shaped lesion, with ultrasound demonstrating a hyperechoic solid mass with indistinct margins in the upper inner quadrant of the left breast. Core needle biopsy and histopathological analysis were performed confirming the diagnosis of benign breast hemangioma, with no further diagnostic workup or treatment indicated.

## Introduction

Breast masses frequently arise in clinical practice, often identified as incidental findings during routine evaluations in primary care settings and screening mammography. Among these masses, vascular tumors, although rare, can present significant challenges in both diagnosis and clinical management. Hemangiomas, the most common benign vascular tumor, are predominantly idiopathic, arising from endothelial cell hyperplasia in blood vessel walls [1]. These tumors are rarely described as affecting the breast and are rarer still in male patients, with only a handful of cases having been published. Breast hemangiomas have a wide range of clinical presentations, from asymptomatic lesions without obvious exam findings to symptomatic palpable abnormalities within the breast parenchyma. This variability in presentation makes initial diagnosis more challenging [1,2].

As with other breast masses, mammography and sonography are first-line imaging modalities used in the diagnosis of these lesions. However, as there are no conclusive imaging findings specific to hemangiomas, further workup with breast MRI or histopathological analysis is often necessary to confirm diagnosis and rule out other potential etiologies, including malignant lesions such as angiosarcomas. Due to the rarity of breast hemangiomas, particularly in male patients, as well as the diagnostic uncertainties regarding the potential for malignancy that may arise during initial workup, surgical excision is often a consideration in definitive management. However, in cases where a diagnosis of benign hemangioma is confirmed, as this report demonstrates, conservative management with observation alone can be employed with an excellent prognosis. Here, we describe a rare breast imaging diagnosis of hemangioma in an equally uncommon symptomatic male patient and underscore the importance of thorough diagnostic workup of male patients.

## Case presentation

**Figure 1:**
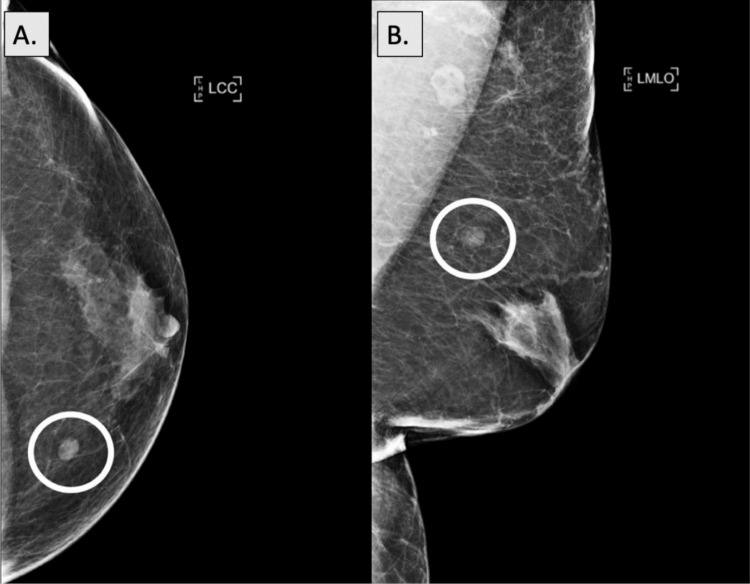
Diagnostic mammography of left breast hemangioma Craniocaudal (A) and mediolateral oblique (B) views from a diagnostic mammogram of the left breast show an isodense, oval mass with circumscribed margins in the upper inner quadrant (white circles). An underlying retroareolar, flame-shaped density is also seen radiating from the nipple, indicative of benign gynecomastia.

**Figure 2:**
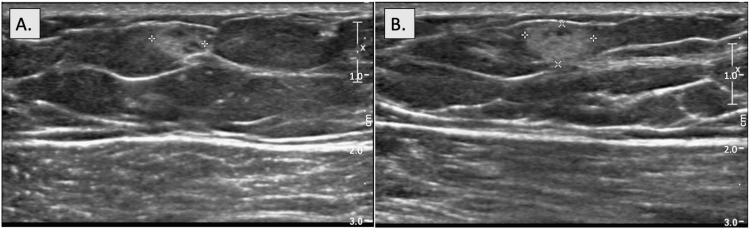
Sonography of left breast hemangioma Sonography performed in the left breast using radial (A) and anti-radial (B) approaches demonstrates an oval, solid mass with parallel orientation and indistinct margins measuring 9x6x7 mm at the 11 o’clock position, located 7 cm from the nipple. Internal echotexture is hyperechoic.

A 69-year-old male patient with no relevant past medical history presented to the primary care clinic with complaints of focal and constant left-sided chest wall pain to palpation. He denied any other associated symptoms or aggravating factors, noting temporary relief with the use of ibuprofen. Physical exam was notable for tenderness to deep palpation of the left breast in the upper inner quadrant, without any palpable masses. The patient was subsequently sent for a chest x-ray, which was unremarkable, and referred for bilateral diagnostic mammography. Mammography revealed an isodense, oval mass with circumscribed margins in the upper inner quadrant of the left breast correlating with the patient’s pain, as well as underlying benign gynecomastia, seen as a flame-shaped density radiating from the nipples bilaterally (Figure 1). Ultrasound further characterized this mass as hyperechoic and solid with parallel orientation and indistinct margins measuring 9x6x7 mm at the 11 o’clock position in the left breast (Figure 2).

**Figure 3:**
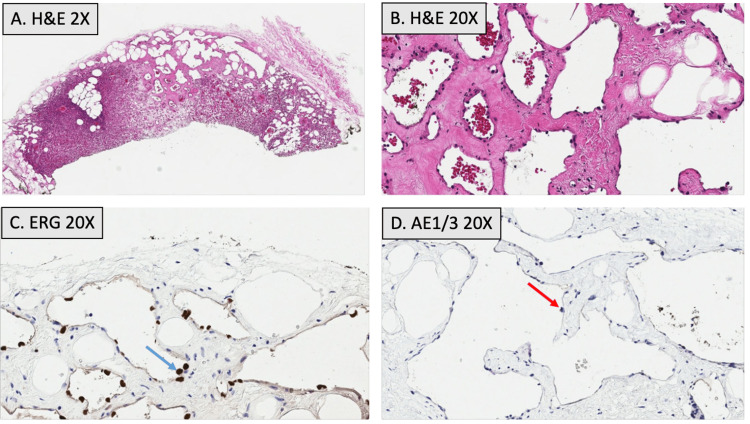
Histopathology of left breast hemangioma Histopathological analysis of the left breast mass shows vascular channels lined by a single layer of flattened endothelial cells with focal adipocytes. No observable endothelial atypia, multilayering, or mitotic activity is seen, both at 2x magnification (A) and 20x magnification (B). Immunostains reveal that the endothelial cells are positive for ETS-related gene (*ERG*) (C, denoted by blue arrow), while negative for pancytokeratin AE1/3 (D, denoted by red arrow).

The patient was subsequently referred for a left-sided ultrasound-guided core needle biopsy of the breast lesion. Histopathology was performed showing a circumscribed vascular lesion composed of back-to-back capillaries and cavernous spaces with focal intervening adipocytes, without evidence of endothelial atypia, multilayering, or mitotic activity (Figure 3A-3B). Immunohistochemical staining was positive for ETS-related gene (*ERG*) and smooth muscle actin, while negative for pancytokeratin AE1/3. These findings were consistent with a diagnosis of benign breast hemangioma (Figure 3C-3D). Due to a low concern for malignancy or malignant transformation, no further workup or surgical intervention was pursued. 

## Discussion

Breast masses are very common and cover a wide range of differential diagnoses, including vascular tumors. Hemangiomas are benign vascular tumors that are idiopathic in etiology with no known risk factors, arising from endothelial cell hyperplasia in blood vessel walls [1]. These tumors are well-documented as occurring in other organs but are rarely described as affecting the breast, with studies citing an overall incidence rate of approximately 0.4%; in fact, a retrospective study of 10,000 breast biopsies over 10 years showed only 0.15% positive diagnoses for hemangiomas [2].

Though the majority of breast masses are found in women of reproductive age, men can also be affected. Male breast masses are most often benign and can typically be attributed to gynecomastia, a common incidental finding affecting both young patients in the pubertal period and elderly patients [3,4]. Gynecomastia may present with focal pain, so should be high on the differential in a male patient presenting with breast tenderness. Nevertheless, other etiologies, both benign and malignant, should be considered and ruled out. While the exact sex distribution of breast hemangiomas is unclear, a previous paper from 2018 cited only 15 published case reports involving male patients [5]. Our review of literature from reports published since 2018 has shown an additional three cases [6-8], making these lesions an exceptionally rare diagnosis in males, likely exacerbated by under-diagnosis in an unlikely patient population.

Breast hemangiomas have variable clinical presentations. Some may be completely asymptomatic while others present as painful and palpable masses within the breast tissue, especially for larger lesions and those located superficially within the subcutaneous tissues [1,2]. Overlying skin changes and discoloration of the breast are also frequent clinical findings based on reported cases [5]. However, as demonstrated in this instance, patients may also present with non-palpable lesions accompanied by vague focal breast pain, and as such, physical exam findings should not necessarily rule out this diagnosis or prevent further workup.

Though there are no pathognomonic imaging findings associated with breast hemangiomas, mammography most commonly reveals oval or lobular masses, well-circumscribed in shape, with variable echogenicity, ranging from high density to isodense lesions when compared to surrounding fibroglandular breast tissue [1-5]. Cavernous hemangiomas, characterized by large, dilated blood vessels, can also uniquely present as complex solid and cystic masses with increased heterogeneity [1], and may even show the presence of fine calcifications on mammography [1-4]. Sonography of breast hemangiomas typically shows superficially located oval masses with parallel orientation and well-circumscribed or lobulated margins, as well as variable internal echotexture, though most are hypoechoic. Hemangiomas are rarely hyperechoic with indistinct margins as demonstrated in this case [1-3]. MRI findings are less robust but have been described as ovoid masses with circumscribed borders, isointense on T1-weighted images, and hyperintense on T2-weighted images [2,9].

The wide range of clinical presentations and imaging findings associated with breast hemangiomas may necessitate confirmation of diagnosis through biopsy and histopathological analysis. Despite the unexpected sonographic findings presented in this case, the histopathological features of the tumor were more consistent with those classically seen in benign hemangiomas, namely the presence of densely-packed small capillaries with fibrous bands and adipocytes, as well as a lack of atypia, multilayering, or mitotic activity indicating potential malignancy [10]. Additionally, the tumor displayed positive immunoreactivity for ERG, a transcription factor with high sensitivity and specificity for vascular lesions [10], with studies showing 100% expression of ERG in endothelia of hemangiomas [11]. Histopathology was also positive for the vascular marker smooth muscle actin, which has been cited as a potential tool in distinguishing benign vascular lesions from malignant tumors [10,12]. Based on these results, the tumor was confidently diagnosed as a benign breast hemangioma.

While hemangiomas are benign tumors with low malignant potential, because of clinical and radiographic overlap with cancerous tumors, including well-differentiated angiosarcomas, the diagnosis of a vascular lesion within the breast may prompt consideration of surgical excision. Furthermore, cases have been reported of benign mimickers of malignancy as well as malignant tumors being initially misdiagnosed as hemangiomas [13-16]. Despite these factors in favor of surgical excision, several studies have shown an overall low incidence of misdiagnosis in vascular lesions sent for analysis following surgical excision and no significant difference in the clinical course of patients with both excised and non-excised lesions [2,10,17,18]. With this evidence in mind, excision may be avoided for lesions without atypia or pathological/clinical discordance as documented here, thereby sparing patients invasive and potentially unnecessary surgical procedures. Given the excellent prognosis of breast hemangiomas and insignificant risk of recurrence or malignant transformation, this patient did not undergo further workup or treatment.

## Conclusions

This case of a breast hemangioma diagnosed in a 69-year-old male patient underscores the rarity of vascular tumors and highlights the potential for symptomatic presentations in an uncommon patient population. Our findings reinforce the importance of considering this diagnosis in male patients presenting with breast pain, with or without palpable lesions, or asymptomatic breast masses. More importantly, the variability in clinical presentations necessitates a thorough diagnostic approach with both imaging studies and histopathological analysis to effectively rule out more dangerous etiologies. While there have been differing recommendations regarding surgical versus nonsurgical management of breast hemangiomas for the purpose of excluding malignancy, lesions without atypia or pathological/radiological discordance may be managed conservatively, as was highlighted in this case.
